# Monitoring Fetal Electrocortical Activity during Labour for Predicting Worsening Acidemia: A Prospective Study in the Ovine Fetus Near Term

**DOI:** 10.1371/journal.pone.0022100

**Published:** 2011-07-15

**Authors:** Martin G. Frasch, Ashley E. Keen, Robert Gagnon, Michael G. Ross, Bryan S. Richardson

**Affiliations:** 1 Department of Obstetrics and Gynecology, Lawson Health Research Institute, The University of Western Ontario, London, Ontario, Canada; 2 Department of Physiology and Pharmacology, Lawson Health Research Institute, The University of Western Ontario, London, Ontario, Canada; 3 Department of Obstetrics & Gynecology, McGill University, Quebec, Canada; 4 Department of Obstetrics and Gynecology, Geffen School of Medicine at the University of California, Los Angeles, Los Angeles, California, United States of America; 5 Department of Obstetrics and Gynecology, CHU Sainte-Justine Research Centre, Université de Montréal, Quebec, Canada; Institute of Clinical Effectiveness and Health Policy, Argentina

## Abstract

**Background:**

Severe fetal acidemia during labour with arterial pH below 7.00 is associated with increased risk of hypoxic-ischemic brain injury. Electronic fetal heart rate (FHR) monitoring, the mainstay of intrapartum surveillance, has poor specificity for detecting fetal acidemia. We studied brain electrical activity measured with electrocorticogram (ECOG) in the near term ovine fetus subjected to repetitive umbilical cord occlusions (UCO) inducing FHR decelerations, as might be seen in human labour, to delineate the time-course for ECOG changes with worsening acidemia and thereby assess the potential clinical utility of fetal ECOG.

**Methodology/Principal Findings:**

Ten chronically catheterized fetal sheep were studied through a series of mild, moderate and severe UCO until the arterial pH was below 7.00. At a pH of 7.24±0.04, 52±13 min prior to the pH dropping <7.00, spectral edge frequency (SEF) increased to 23±2 Hz from 3±1 Hz during each FHR deceleration (p<0.001) and was correlated to decreases in FHR and in fetal arterial blood pressure during each FHR deceleration (p<0.001).

**Conclusions/Significance:**

The UCO-related changes in ECOG occurred in advance of the pH decreasing below 7.00. These ECOG changes may be a protective mechanism suppressing non-essential energy needs when oxygen supply to the fetal brain is decreased acutely. By detecting such “adaptive brain shutdown,” the need for delivery in high risk pregnant patients may be more accurately predicted than with FHR monitoring alone. Therefore, monitoring fetal electroencephalogram (EEG, the human equivalent of ECOG) during human labour may be a useful adjunct to FHR monitoring.

## Introduction

Studies of electrocortical activity in the ovine fetus near term using electrocorticogram (ECOG) recordings reveal alternating epochs of low-voltage/high frequency (LV/HF) electrocortical activity with high-voltage/low frequency (HV/LF) ECOG indicative of behavioural states that relate to cortical neuronal activity [1]–[3] (reviewed in [4]). This ECOG state activity becomes altered in response to induced hypoxia both chronically by reducing maternal inspired oxygen over several hours [5] and acutely by occluding the umbilical circulation over several minutes [6], with disruption in behavioural state cyclicity and flattening of the ECOG voltage amplitude. These ECOG changes are paralleled by changes in cerebral metabolism. An overall decrease in oxygen uptake and increasing reliance on anaerobic metabolism are likely triggered by critical decreases in the brain's oxygenation [6], [7].

In studies of cerebral hypoxic-ischemia in adults, there is a reproducible sequence of changes in cerebral metabolism [8]. Below an upper ischemic flow threshold suppression of synaptic transmission occurs, but neuronal energy levels and cellular integrity are maintained. When a lower ischemic flow threshold is reached, membrane failure occurs that indicates energy depletion and is closely associated with the development of structural cell damage. Hence, the initial suppression of synaptic transmission with a flattening of ECOG as a response to worsening hypoxia may be protective by decreasing the energy consumed by Na^+^-K^+^-ATPase involved in the generation of synaptic potentials [8]. Importantly, these ECOG and related metabolic changes within the brain should occur in advance of asphyxia-mediated brain injury. Thus, changes in ECOG might prove useful for signaling a risk of impending brain injury.

Electronic fetal heart rate (FHR) monitoring is the mainstay for assessing fetal health during labour, because the absence of FHR decelerations is highly predictive of normal fetal blood gas and pH at birth [9]–[12]. Variable-type FHR decelerations due to umbilical cord compression with acute reduction in fetal oxygenation are the most common decelerations observed during labour in humans [13]. When frequent and/or severe, these FHR patterns are associated with an increased incidence of concerning acidemia in the newborn [14]–[16]. However, FHR monitoring is less predictive of the degree of hypoxia-induced acidemia, *i.e.*, FHR monitoring has a low positive predictive value for clinically important metabolic acidosis at birth. Accordingly, the study of the ancillary monitoring techniques was recommended [12]. Over the past decade several complimentary monitoring techniques were assessed including computerized FHR data acquisition and interpretation, fetal pulse oximetry and ECG waveform analysis [9]–[11]. To date, none of these techniques have become ‘standard of care’ in North America. Hence, there is a clinical need to improve the existing technologies used for monitoring fetal health during labour.

We hypothesized that consistent changes in fetal ECOG will occur well in advance of attaining a severe degree of hypoxic-acidemia due to suppression of neuronal synaptic activity below the upper ischemic flow threshold. We studied ECOG in the near term ovine fetus, a well established model of human pregnancy, subjected to repetitive umbilical cord occlusions (UCO) leading to FHR decelerations with worsening acidemia as might be seen in human labour. Animals were studied through a series of mild, moderate and severe UCO until fetal arterial pH fell below 7.00, this being the threshold for severe acidemia in the human newborn and fetal sheep below which there is increasing risk of hypoxic-ischemic brain injury [17]–[22]. Changes in ECOG amplitude and frequency components were analyzed in relation to the degree of fetal acidemia as well as the associated cardiovascular responses. Confirming our hypothesis we found changes in ECOG occurring in advance of the threshold for severe acidemia with pH<7.00. This finding has clinical importance, since the feasibility of recording the electroencephalogram (EEG, the human equivalent of ECOG) from a scalp electrode during human labour has been demonstrated [23], [24].

## Results

### Arterial blood gases and pH

epetitive UCO insults resulted in fetal arterial blood gas, oxygen saturation (O_2_Sat) and pH changes with each cord occlusion as well as cumulative changes over the course of the study as reported (Table 1, [25], [26]). The key finding relevant to interpreting the cardiovascular and ECOG responses herein presented is that fetal arterial pH values showed a progressive decrease throughout the UCO series from baseline values of 7.36±0.01 to 6.90±0.04 following completion of the severe UCO series (p<0.05). Two animals reached the target pH<7.00 during the moderate UCO series, while the remaining eight animals took between 20 and 100 minutes during the severe UCO series to reach the target pH. The animal with the lowest arterial pH (6.64) died shortly after stopping the repetitive cord occlusion insults.

**Table 1:** Fetal arterial blood gas, O_2_ Sat and pH measurements.

	pO_2_ mmHg	pCO_2_ mmHg	O_2_ Sat %	pH
Baseline	18.2±.8	52.7±.9	50.0±3.1	7.36±.01
Mild UCO series				
1^st^ UCO	9.7±1.4*	57.3±1.7*	18.3±4.6*	7.32±.02
5 min post	18.5±.9	52.8±1.0	47.2±3.5	7.32±.03
Moderate UCO series				
1^st^ UCO	10.8±1.0*	57.0±1.5*	19.7±3.7*	7.28±.03*
5 min post	19.0±.8	56.2±1.3	43.5±3.9	7.19±.04*
Severe UCO series				
1st UCO	10.8±1.9*	58.1±1.4*	16.4±4.1*	7.21±.03*
5 min post	19.4±1.8	77.3±9.3*	27.2±3.2*	6.90±.04*
Recovery				
1^st^ hr	17.4±.7	48.9±1.0	34.8±2.2*	7.18±.02*

Values are means ± SEM. N = 10. UCO = umbilical cord occlusion.

*, p<0.05 vs. baseline.

### Cardiovascular responses

During the baseline control period, FHR averaged 163±5 bpm. While variably increased during the 10 minute periods without UCO after the mild and moderate UCO series and through the first hour of recovery after the severe UCO series, none of these changes were significant (Table 2). During each of the UCO series the FHR decelerations were of similar degree reaching an average nadir of 80±2 bpm. Consequently, the UCO-related depth of FHR decelerations averaged 83±2 bpm (Table 2).

**Table 2:** Fetal cardiovascular measurements.

	Mean FHR, bpm	Mean ABP, mmHg	FHR_nadir_, bpm	ABP_max_, mmHg	ABP at FHR_nadir_, bpm
Baseline	163±5	45±4			
Mild UCO series			83±5*	64±3*	67±8*
Post Mild UCO series	185±14	48±4			
Mod UCO series			78±5*	63±4*	54±4*
Post Mod UCO series	171±11	59±7			
Severe UCO series			78±4*	65±6*	60±10*
Recovery 1^st^ hr	189±7	57±11			

Values are means ± SEM. N = 10. UCO = umbilical cord occlusion; FHR = fetal heart rate (bpm, beats per minute); ABP = arterial blood pressure, mmHg; FHR_nadir_ = UCO-related nadir of FHR deceleration; ABP_max_, = maximum ABP during UCO; ABP_FHR nadir_ = ABP at FHR_nadir_;

*, p<0.05 vs. baseline.

Likewise, during the baseline control period, the ABP averaged 45±4 mmHg. The maximum ABP during UCO was of a similar degree on average for each of the UCO series at 64±1 mmHg, resulting in a UCO-related maximal ABP increase of 19±1 mmHg (Table 2). For the mild UCO series, the ABP we measured at the time of the nadir of the FHR deceleration (ABP_FHR nadir_) was higher on average than the respective maximum ABP during UCO (ABP_max_) at 67±8 *vs.* 64±3 mmHg (Table 2). For the moderate and severe UCO series, ABP_FHR nadir_ was now lower on average than the respective ABP_max_, at 54±4 *vs.* 63±4 mmHg and at 60±10 *vs.* 65±6 mmHg (Table 2). That is, while the maximum increase in ABP during UCO was similar for the three UCO series, the increased ABP was not sustained and fell somewhat when again measured at FHR_nadir_ for the moderate and severe UCO series. Accordingly, we found no correlation of ABP_max_ or maximal ABP increase (ΔABP_max_) to fetal pH as assessed for the 10 minute intervals prior to blood sampling during the UCO series for each animal. However, ABP_FHR nadir_ was found to decrease and ΔABP_FHR nadir_ was found to increase with lower pH, R = 0.45 (p = 0.01) and R = −0.52 (p<0.01), respectively, again indicating an inability to sustain the UCO-related hypertension with worsening acidemia.

### Cerebral electrical responses (ECOG)

During the baseline control period, mean ECOG amplitude averaged 88±13 µV, and did not change significantly as measured through each of the UCO series (Table 3). Conversely, mean ECOG 95% SEF which averaged 14.4±0.4 Hz during the baseline control period, was significantly decreased as measured through the moderate and severe UCO series at 11.4±0.6 Hz and 9.6±0.4 Hz, respectively (both p<0.05) (Table 3). ECOG amplitude and 95% SEF as measured during the UCO-induced FHR decelerations were little changed with the mild UCO series, but were significantly decreased with the severe UCO series at 60±12 µV and 9.9±1.1 Hz, respectively, when compared to the respective baseline values (both p<0.05) (Table 3). Likewise, during the severe UCO-induced FHR decelerations, ECOG amplitude was markedly decreased from that for the 30 seconds prior to these UCO by 54±13 µV, although there was no related change in the ΔECOG SEF (Table 3).

**Table 3:** ECOG measurements.

	Mean AMP, µV	Mean 95% SEF, Hz	UCO AMP, µV	UCO 95% SEF, Hz	UCO ΔAMP, µV	UCO ΔSEF, Hz
Baseline	88±13	14.4±0.4				
Mild UCO series	97±14	13.0±0.7	90±27	14.1±2.1	9±7	−0.8±0.9
Moderate UCO series	98±24	11.4±0.6*	81±21	11.2±0.9	15±8	−0.5±1.2
Severe UCO series	98±19	9.6±0.4*	60±12*	9.9±1.1*	54±13^+^	−1.4±2.0
Recovery 1^st^ hr	91±10	13.8±0.4				

Values are means ± SEM. N = 8. UCO = umbilical cord occlusion; AMP = amplitude; 95% SEF = 95% spectral edge frequency; UCO AMP and 95% SEF = respective ECOG measurements during UCO; ΔUCO AMP and SEF = respective ECOG measurement change from 30 seconds prior to each UCO to that during UCO, ^+^ p<0.05 for ECOG amplitude differences during the severe UCO series 30 s prior to the UCO to the amplitude during the UCO;

*p<0.05 vs. baseline.

For individual animals, mean ECOG amplitude during the UCO-induced FHR decelerations also decreased with worsening acidemia and lower pH as assessed at 10 minute intervals prior to each blood sampling during the UCO series, R = 0.51 (p<0.01). There was no significant relationship between mean ECOG SEF and fetal pH change when similarly analyzed. Additionally, ΔECOG AMP and ΔECOG SEF, *i.e.*, the relative change in ECOG amplitude and 95% SEF prior versus during the UCO-induced FHR decelerations, were both seen to increase with worsening acidemia and lower pH, R = −0.65 and −0.68, respectively (both p<0.001).

**Figure 1:**
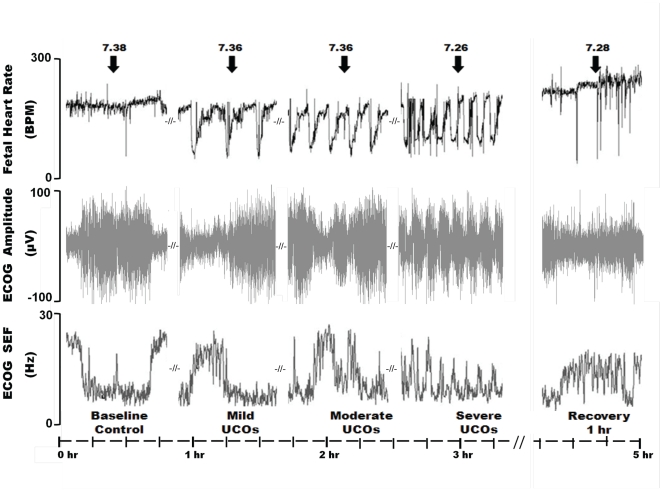
Representative segments of fetal heart rate (BPM, beats per minute) and electrocorticogram (ECOG). Umbilical cord occlusion (UCO) induced changes are shown in 15 minutes segments of FHR, ECOG amplitude (µV) and 95% spectral edge frequency (SEF, Hz). Baseline and the 1^st^ hour of recovery are shown for comparison. Note an early emergence of the time correlated changes in FHR decelerations and increases in ECOG SEF with modest fetal acidemia (pH = 7.26). The arrows indicate fetal blood sampling and the corresponding pH values at these time points.

**Figure 2:**
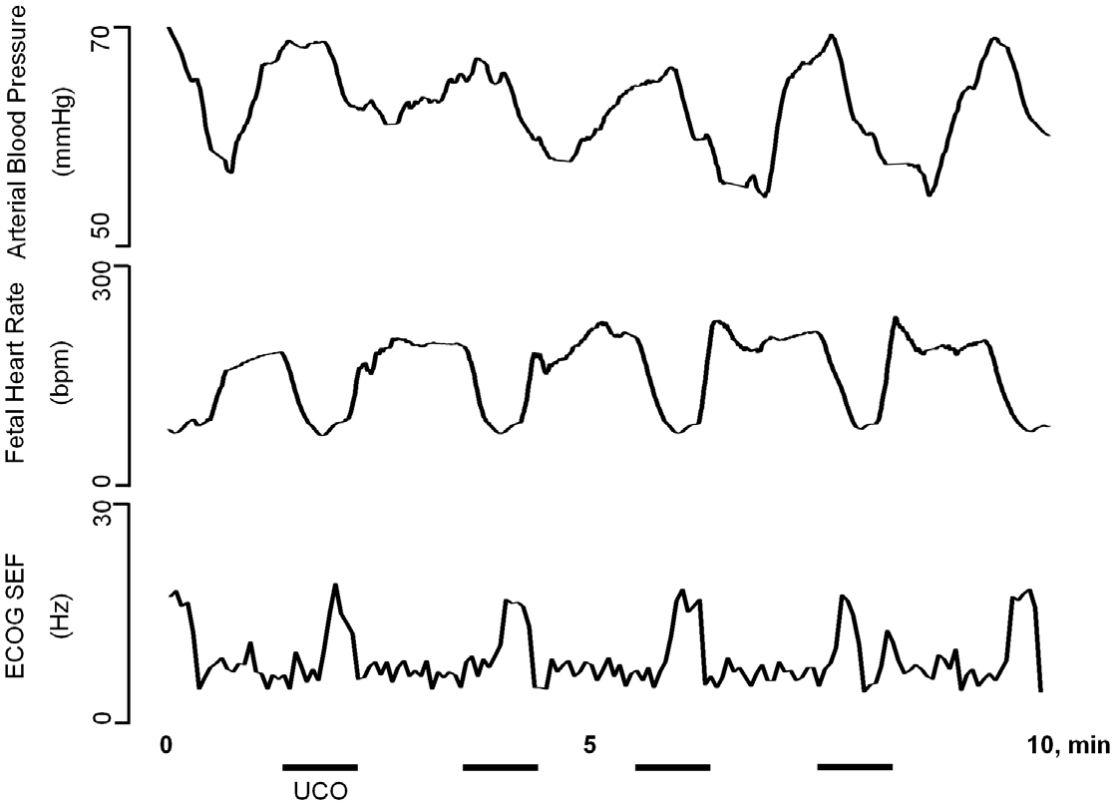
Representative segments of correlated changes in fetal heart rate decelerations (BPM, beats per minute) and electrocorticogram (ECOG) spectral edge frequency (SEF, Hz) spiking pattern. ECOG SEF, FHR and fetal arterial blood pressure (ABP, mmHg) are shown during a 10 minutes segment demonstrating the correlated changes in FHR decelerations and ECOG SEF spiking pattern during each umbilical cord occlusion (UCO, indicated by the black bars). Note pathological decreases of ABP during the FHR decelerations.

In the course of worsening UCO and associated acidemia we observed a specific pattern of change in ECOG SEF in relation to FHR decelerations for all of the animals studied (Figures 1 and 2). This pattern was visually recognized and defined by the onset of abrupt increases in ECOG 95% SEF values up to 23±2 Hz towards the end of each UCO-induced FHR deceleration. This pattern usually lasted no more than 15 to 20 seconds before rapidly declining to 3±1 Hz between FHR decelerations. This SEF ‘spiking’ pattern was detected 52±13 minutes (range 1 h 58 min to 25 min) prior to reaching the target pH<7.00. Once initiated, this pattern continued in all animals until the cord occlusion insults were stopped. However, the onset of this pattern in relation to worsening acidosis was variable occurring at a pH of 7.24±0.04 on average, but ranging from 7.36 to 7.06 as determined from the most proximate blood sample (outlined in Methods). Of note, the onset of this pattern was closely related to the inability to sustain the UCO-related increase in ABP; ABP was now either unchanged or decreased as measured at the nadir of each FHR deceleration when compared to pre-UCO values. Accordingly, for individual animals the lengths of time over which the ECOG SEF ‘spiking’ pattern and the hypotensive ABP responses were seen prior to reaching the target pH<7.00 were similar and highly correlated, R = 0.89 (p<0.001). Most animals showed the UCO related spikes in ECOG SEF slightly before showing the ABP_FHR nadir_ hypotensive response.

## Discussion

The main finding of this study is the detection of an ECOG pattern occurring in advance of worsening fetal acidemia and associated with FHR decelerations induced by repetitive umbilical cord occlusions as they may occur during human labour. Monitoring human fetal EEG during labour may potentially be an ancillary tool to improve the ability to detect worsening fetal acidemia in advance of brain injury. We propose that the pathophysiological mechanism producing the observed ECOG pattern is an acute adaptive response of the fetal brain to suppress non-essential energy needs during asphyxia. We discuss the correlated changes in arterial blood gases, cardiovascular responses and ECOG in this context.

### Arterial blood gases and pH

All fetuses attained a severe metabolic acidemia with base excess values of −16.6±1 mmol/L as reported [25], [26]. However, the degree of fetal acidemia after each of the UCO series differed somewhat amongst the animals as did the actual severity of acidemia attained. Accordingly, we analyzed the mean cardiovascular and ECOG findings for each of the UCO series, as well as individual animal findings for the 10 minute intervals prior to each blood sampling during the UCO series to further assess changes with worsening acidosis.

### Cardiovascular responses

Cord occlusions of one minute duration during mild UCO series resulted in an immediate fall in FHR to a nadir value of 83±5 bpm at or shortly after the end of the occlusion and this did not change significantly in magnitude during successive occlusions throughout the UCO series. This finding is similar to that of de Haan et al. [27] with their study of repetitive UCO of 1 minute duration every 2.5 minutes in fetal sheep, and indicates that the chemoreflex mechanisms shown to mediate fetal bradycardia with acute hypoxia are consistently active despite severe acidosis [28]–[30].

Fetal ABP increased during each UCO to a maximum value of 64±1 mmHg or by 19±1 mmHg on average from baseline values, and this again did not change significantly in magnitude throughout the UCO series. This finding was also noted by de Haan et al. [27] and suggests that the chemoreflex mechanisms leading to peripheral vasoconstriction are also intact despite severe fetal acidemia and augment the initial hypertension with mechanical occlusion of the umbilical arteries [28]–[30]. However, this UCO-related increase in ABP was not sustained as indicated by the lower ABP_FHR nadir_ values at the end of each UCO or shortly thereafter during the moderate and severe UCO series, and with the fall in ABP_FHR nadir_ values well correlated with the degree of fetal acidemia. This biphasic blood pressure pattern was likewise noted by de Haan et al. [27]. After the initial UCO-related increase in ABP they observed a gradual progressive fall with the minimum blood pressure usually occurring shortly after release of the occluder, and the recovery time to regain normal blood pressure values was prolonged in the face of worsening acidemia. The onset and progression of this biphasic blood pressure response may indicate an inability to maintain the chemoreflex mediated vasoconstriction or an inability to maintain cardiac output due to impaired myocardial contractility, or both, as shown in both humans and sheep with fetal acidemia [31], [32].

### Cerebral electrical responses: mechanisms of changes in ECOG amplitude and frequency

Mean ECOG amplitude as measured through each of the mild, moderate and severe UCO series was marginally increased but not significantly different from that of the baseline control value. This can be attributed to an overall increase in time spent in HV/LF state activity as well as indeterminate voltage/frequency (IV/F) activity, but then offset by a variable decrease in voltage amplitude during each UCO in the near term ovine fetus with repetitive short duration UCO insults [6], [33], [34].

Mean ECOG amplitude during the UCO-related FHR decelerations was variably decreased while the change from that 30 seconds prior to each UCO, *i.e.*, ΔECOG AMP, was conversely increased. However, this was only significant for the severe UCO series. This is consistent with an adaptive suppression of synaptic transmission activity to decrease energy needs with longer UCO insults [5], [6], [33], [35]. This adaptive metabolic shutdown by the ovine fetal brain appears to be mediated by endogenous activation of adenosine A1 receptors during critical decreases in oxygenation as shown by Hunter et al. with cord occlusion study during adenosine A1 receptor blockade [5]. The onset of this response is rapid — within 30 to 60 seconds after complete cord occlusion — whether measured by decreased ECOG amplitude or cerebral metabolic rate [5], [6], [33], [35]. However, this response will also depend on the integrity of other defense mechanisms including the ability to redistribute and maintain increased blood flow to the brain. As such, the significant decrease in ECOG amplitude during severe UCO in the present study may be explained by the related hypertensive-hypotensive blood pressure pattern, which would serve to limit the hypoxia-induced increases in cerebral blood flow [6], [7], [36]. The decrease in ECOG amplitude during UCO and the increase in ΔECOG AMP showed a modest relationship to falling arterial pH with R values of 0.51 and −0.65, respectively. This finding is again similar to that of de Haan et al. in their study of repetitive UCO where fetal ECOG intensity decreased continuously with worsening acidemia [34].

Mean ECOG 95% SEF during each of the UCO series decreased in a stepwise manner from the baseline control value consistent with increasing time spent in HV/LF and IV/F activity, but again offset by the abrupt increases in SEF values towards the end of each UCO-induced FHR deceleration during the moderate and/or severe UCO with worsening acidemia. Of note, a study by Thaler et al. [24] using SEF analysis of fetal EEG during human labour with variable FHR decelerations but without acidemia showed voltage amplitude to be increased while SEF was decreased — consistent with a transition to HV/LF state activity, albeit in a limited number of healthy subjects.

Mean ECOG 95% SEF during the UCO-related FHR decelerations was similar to that measured through each of the respective UCO series and thereby also decreased in a stepwise manner from the baseline control values, but with no significant change from that measured 30 seconds prior to each UCO, *i.e.*, ΔECOG SEF. This is again consistent with increasing time spent in HV/LF and IV/F activity with worsening UCO insults. However, with worsening UCO and fetal acidemia we also noted the onset of abrupt increases in SEF of short duration towards the end of each UCO-induced FHR deceleration in all animals. This SEF ‘spiking’ pattern was well correlated with worsening of the biphasic hypertensive-hypotensive blood pressure response. The appearance of the SEF ‘spiking’ pattern is likely triggered by a further limitation in the hypoxia-induced increases in cerebral blood flow due to the worsening hypotension or worsening acidemia. The emergence of this SEF ‘spiking’ pattern suggests that neuronal activities are being differentially affected and may reflect the relative dominance of neocortical γ-aminobutyric acid (GABA)ergic inhibitory interneuron activity that is capable of high frequency oscillations [37]. This might occur due to the active participation in suppression of synaptic transmission by excitatory neurons, or due to hierarchal ‘shutdown’ of excitatory neurons in advance of inhibitory interneurons when cerebral oxygenation becomes rate-limiting for metabolic activity, since GABAergic signaling is less energy demanding [38]. The appearance of the SEF ‘spiking’ pattern with worsening acidemia and the increase in ΔECOG SEF with falling pH (as assessed for the 10 minutes prior to each blood sampling during the UCO series) also support a link between ECOG alterations and systemic acidemia. However, the ΔECOG SEF-pH relationship was modest, with an R value of −0.68, and the onset of the SEF ‘spiking’ pattern in relation to worsening acidosis was variable ranging from pH 7.36 to 7.06 and appearing as early as 1 h 58 min to 25 min prior to attaining the target pH<7.00.

### The adaptive cardiovascular and ECOG responses to worsening acidemia are individually specific, and likely dependent on previous hypoxic-asphyxic exposures

Despite the similar UCO insults, animals showed differing time courses for metabolic deterioration and for the UCO-related changes in ECOG. Similar findings were noted by de Haan et al. in their study of repetitive UCO [27], [34]. They reported a relationship to cardiovascular deterioration as reflected by onset and progressive worsening in the biphasic hypertensive-hypotensive blood pressure response. This could involve the vasoconstrictor response to acute hypoxia and thereby the ability to redistribute blood flow to the brain, which has been shown to be diminished in the ovine fetus with prior hypoxic exposure [39]. With repetitive UCO as studied, a diminished redistribution of blood flow to the brain might lead to earlier onset of UCO-related changes in ECOG, while a diminished redistribution of blood flow away from carcass tissues might slow the buildup of lactic acid and thereby mitigate the fall in systemic pH. While these individual differences should not alter the principal observation of the reported ECOG pattern with repetitive UCO and worsening acidemia, the time course for emergence of this pattern requires further study in advance of clinical application.

### Cerebral electrical responses (ECOG): translational implications

Overall, our data provide support for a link between ECOG alterations and systemic acidemia in the ovine fetus near term subjected to repetitive cord occlusions. Cerebral adaptive circulatory and metabolic mechanisms are capable of rapidly suppressing synaptic transmission to reduce non-essential energy needs when oxygen supply to the fetal brain is decreased acutely [5], [6], [33], [35]. In this regard, the animals in the present study showed a rapid normalization of ECOG during the recovery period except for the animal with the lowest pH, which died. The fact that we found no evidence of seizure-like activity strongly suggests that the observed ECOG changes were adaptive rather than reflecting evolving cerebral injury.

Although individual differences in emergence of the SEF ‘spiking’ pattern were seen, the main clinical implication of our findings remains that the UCO-related changes in ECOG SEF occurred well in advance of the threshold for severe acidemia at which increased risk for asphyxia-mediated brain injury is reported [17]–[21], [40]. Given the technologic advances for monitoring fetal EEG during human labour, fetal EEG may be a useful adjunct to electronic FHR monitoring for signaling ‘adaptive metabolic shutdown’ of the fetal brain, and thereby the need for delivery in high risk pregnant patients [24].

## Materials and Methods

### Surgical Preparation

Ten near term (125±1 days gestation) fetal sheep of mixed breed were surgically instrumented (term = 145 days, Table 4). The anesthetic and surgical procedure and postoperative care of the animals were performed as published [41]. Anesthesia was induced with an injection of sodium thiopental (1 g in 40 mL solution; Abott Laboratories Ltd., Montreal, Canada) into the maternal jugular vein and maintained throughout surgery with 1–1.5% isoflurane in oxygen (Halocarbon Laboratories, Hackensack, NJ). Using sterile technique under general anesthesia, a midline incision was made in the lower abdominal wall, and the uterus was palpated to determine fetal number and position. The upper body of the fetus and proximal portion of the umbilical cord were exteriorized through an incision in the uterine wall. Polyvinyl catheters were placed in the right and left brachiocephalic arteries and advanced into the ascending aorta as well as the right brachiocephalic vein and advanced into the superior vena cava. Stainless steel electrodes were implanted over the sternum to record electrocardiographic (ECG) activity. To record ECOG, the stainless steel ECOG electrodes were implanted biparietally on the dura through small burr holes in the skull bone ∼2 mm in diameter made with a hand drill. These burr holes were placed ∼1–1.5 cm lateral to the junction of the sagittal and lambdoid sutures with care taken to avoid puncturing the dura. The bared portion of the wire to each electrode was rolled into a small ball and then inserted into each burr hole to rest on the dura with a small plastic disk then covering each burr hole. This plastic disk was then thinly coated with tissue adhesive (cyanoacrylate) and held in position against the skull bone surrounding each burr hole until firmly adherent. The reference electrode was then placed in the loose connective tissue in the midline overlying the occipital bone at the back of the skull such that the bared wire beyond the knot was embedded. An inflatable silicone occluder cuff was positioned around the proximal portion of the umbilical cord and secured to the abdominal skin. Once the fetus was returned to the uterus, a catheter was placed in the amniotic fluid cavity and another in the maternal femoral vein. Antibiotics were administered intravenously to the mother (0.2 g trimethoprim and 1.2 g sulfadoxine, Schering Canada Inc., Pointe-Claire, Canada) and the fetus and into the amniotic cavity (1 million IU penicillin G sodium, Pharmaceutical Partners of Canada, Richmond Hill, Canada). Amniotic fluid lost during surgery was replaced with warm saline. The uterus and abdominal wall incisions were sutured in layers and the catheters exteriorized through the maternal flank and secured to the back of the ewe in a plastic pouch.

**Table 4:** Subject information.

	Number of fetuses	Weight (kg)	Gender
	1	3.1	Male
	2	1.9	Male
	1	2.7	Male
	2	2.3	Female
	1	3.6	Female
	1	2.2	Female
	1	2.8	Female
	1	2.8	Male
	1	3.3	Female
	1	3.3	Female
Mean±SEM	1.2±0.1	2.8±0.2	

Animals were allowed a 3–4 day postoperative period prior to experimentation. During this period, postoperative daily antibiotic prophylaxis was continued: 1) to the ewe via maternal femoral catheter (6 cc Trivetrin, Schering-Plough, Kenilworth, NJ) and 2) to the fetus via the brachiocephalic vein and amniotic cavity (1,000,000 IU penicillin G sodium, Pharmaceutical Partners of Canada, Richmond Hill, Canada). Arterial blood was sampled for evaluation of maternal and fetal condition and catheters were flushed with heparinized saline to maintain patency. 10,000 USP heparin units were dissolved in 250 cc isotonic NaCl. Animal care followed the guidelines of the Canadian Council on Animal Care and was approved by the University of Western Ontario Council on Animal Care.

### Experimental protocol

Animals were studied through a 1 to 2 hour baseline period, an experimental period of repetitive UCO with worsening acidemia, and were then allowed to recover overnight. The fetal arterial and amniotic pressures, ECOG and ECG were monitored continuously through the control and experimental periods, and first hour of the recovery period.

After the baseline period which began at ∼0800, repetitive UCO were performed with increasing frequency until severe fetal acidemia was detected (arterial pH<7.00), at which time the UCO were stopped. Complete UCO was induced by inflation of the occluder cuff with ∼5 mL saline solution, the exact volumes having been determined by visual inspection and testing at the time of surgery for each animal. During the first hour a mild UCO series was performed consisting of cord occlusion lasting for 1 minute and repeating every 5 minutes. During the second hour a moderate UCO series was performed consisting of cord occlusion for 1 minute duration and repeating every 3 minutes. During the third hour a severe UCO series was performed consisting of cord occlusion for 1 minute duration, repeated every 2 minutes, and this series was continued until the targeted fetal arterial pH was attained. Following the mild as well as the moderate UCO series 10 minute periods with no UCO were undertaken, during which fetal arterial blood was sampled and arterial blood pressure, ECOG, and ECG data were recorded in the absence of fetal heart rate decelerations. After attaining the targeted fetal arterial pH of <7.00 and stopping the repetitive UCO, animals were allowed to recover for ∼24 hours.

Fetal arterial blood samples were obtained during the baseline period (3 mL), at the end of the 1^st^ UCO of each UCO series (1 mL), and ∼5 minutes after each UCO series (3 mL). In addition, fetal arterial blood samples were obtained between UCO at ∼20 and 40 minutes of the moderate and severe UCO series (1 mL), and at 1, 2 and 24 hours of recovery (3 mL). Maternal venous blood samples were also obtained during the baseline period, and at 1 and 24 hours of recovery (3 mL). All fetal arterial blood samples were analyzed for blood gas values, pH, and O_2_Sat with an ABL-725 blood gas analyzer (Radiometer Medical, Copenhagen, Denmark) with temperature corrected to 39.0°C. The amount of blood withdrawn from each fetus was replaced with maternal blood at the end of day 1.

After the 24 hour recovery blood sample, the ewe and the fetus were killed by an overdose of barbiturate (30 mg sodium pentobarbital IV, MTC Pharmaceuticals, Cambridge, Canada) and a post mortem was carried out during which fetal gender and weight were determined, and the location and function of the umbilical cord cuff was confirmed. The fetal brain was then perfusion-fixed with 500 mL of cold saline followed by 500 mL of 4% paraformaldehyde and processed for histochemical analysis as reported [25].

### Data acquisition and analysis

Arterial and amniotic pressures were measured continuously using Statham pressure transducers (P23 ID; Gould Inc., Oxnard, CA). Arterial blood pressure (ABP) was determined as the difference between instantaneous values of arterial and amniotic pressures. A PowerLab system was used for data acquisition and analysis (Chart 5 For Windows, ADInstruments Pty Ltd, Castle Hill, Australia). Arterial and amniotic pressures, ECG and ECOG were recorded and digitized at 1000 Hz. For ECG, a 60 Hz notch filter was applied, while for ECOG, a band pass 0.3–30 Hz filter was used. FHR was triggered and calculated online from arterial pressure systolic peaks.

Average values of FHR and ABP were calculated from recordings through the baseline period, the 10 minute periods without UCO after the mild and moderate UCO series, and through the first hour of recovery after the severe UCO series as reported [26]. For each UCO we calculated the related nadir of the FHR deceleration (FHR_nadir_) and the maximum ABP (ABP_max_) during that UCO, as well as the ABP at the nadir of the FHR deceleration (ABP_FHR nadir_). The UCO - related depth of FHR deceleration (ΔFHR) and maximal ABP increase (ΔABP_max_) as well as the ABP change at the nadir of the FHR deceleration (ΔABP_FHR nadir_) were then calculated as the change from that animal's respective baseline period values. Averaged values for these UCO-related FHR and ABP changes were then determined for each animal for each UCO series as well as for the 10 minute intervals prior to each blood sampling during the UCO series.

Prior to the ECOG analysis, the ECOG signal was sampled down to 100 Hz. Subsequently, the voltage amplitude and 95% spectral edge frequency (SEF, the ECOG frequency below which 95% of ECOG spectral power is found), were calculated over 4 s intervals (Spektralparameter, GJB Datentechnik GmbH, Langewiesen, Germany). For each animal, average values for ECOG amplitude and 95% SEF were then determined from recordings through the baseline period, each of the UCO series and the first hour of recovery. For each UCO-induced FHR deceleration, we also determined the related ECOG amplitude and 95% SEF (*i.e.*, that occurring from the onset of the deceleration to approximately 60 seconds thereafter). Relative changes in ECOG amplitude and 95% SEF were also determined (ΔECOG AMP and ΔECOG SEF) as the differences between the mean ECOG amplitude and 95% SEF during the 30 seconds prior to each UCO and the mean UCO-induced FHR deceleration values. Average values for these ECOG measurements during cord occlusions were then determined for each animal for each UCO series as well as for the 10 minute intervals prior to each blood sampling during the UCO series. The 10 minute interval ECOG values were then correlated to the measured pH values to better delineate the utility of fetal ECOG for predicting metabolic deterioration with repetitive UCO and worsening acidemia.

### Statistical analyses

Normal data distribution was tested using Kolmogorov-Smirnov test. Blood gas, O_2_Sat and pH measurements in response to repetitive cord occlusions were compared to baseline values by one way repeated measures ANOVA with Bonferroni correction for multiple comparisons. FHR, ABP, and ECOG values in response to cord occlusions were analyzed by Friedman repeated measures ANOVA on ranks with Student-Newman-Keuls correction for multiple comparisons. Pearson or Spearman correlation analysis were performed as appropriate and R values are presented where p <0.05 (SigmaStat, Systat Software, Inc., San Jose, Ca). All values are expressed as means ± SEM. Statistical significance was assumed for p <0.05. Not all measurements were obtained for each animal for all time points due to catheter and/or ECOG electrode difficulties as well as differences in the inter-animal rates of deterioration and recovery from UCO (see Results).
